# Prenatal Bisphenol A Exposure and Child Behavior in an Inner-City Cohort

**DOI:** 10.1289/ehp.1104492

**Published:** 2012-04-27

**Authors:** Frederica Perera, Julia Vishnevetsky, Julie B Herbstman, Antonia M Calafat, Wei Xiong, Virginia Rauh, Shuang Wang

**Affiliations:** 1 Department of Environmental Health Sciences, Mailman School of Public Health, and; 2 Columbia Center for Children’s Environmental Health, Columbia University, New York, New York, USA; 3 National Center for Environmental Health, Centers for Disease Control and Prevention, Atlanta, Georgia, USA; 4 Department of Biostatistics, Mailman School of Public Health, and; 5 The Heilbrunn Department of Population and Family Health, Columbia University, New York, New York, USA

**Keywords:** bisphenol A, child behavior, Child Behavior Checklist, prenatal exposure, sex-specific effects

## Abstract

Background: Experimental laboratory evidence suggests that bisphenol A (BPA), an endocrine disruptor, is a neurodevelopmental toxicant. However, there have been limited and inconclusive results with respect to sex-specific BPA effects on child behavior.

Objective: We examined the association between prenatal BPA exposure and child behavior, adjusting for postnatal BPA exposure and hypothesizing sex-specific effects.

Methods: We followed African-American and Dominican women and their children from pregnancy to child’s age 5 years, collecting spot urine samples from the mothers during pregnancy (34 weeks on average) and from children between 3 and 4 years of age to estimate BPA exposure. We assessed child behavior between 3 and 5 years of age using the Child Behavior Checklist (CBCL) and used generalized linear models to test the association between BPA exposure and child behavior, adjusting for potential confounders.

Results: The analysis was conducted on 198 children (87 boys and 111 girls). Among boys, high prenatal BPA exposure (highest quartile vs. the lowest three quartiles) was associated with significantly higher CBCL scores (more problems) on Emotionally Reactive [1.62 times greater; 95% confidence interval (CI): 1.13, 2.32] and Aggressive Behavior syndromes (1.29 times greater; 95% CI: 1.09, 1.53). Among girls, higher exposure was associated with lower scores on all syndromes, reaching statistical significance for Anxious/Depressed (0.75 times as high; 95% CI: 0.57, 0.99) and Aggressive Behavior (0.82 times as high; 95% CI: 0.70, 0.97).

Conclusion: These results suggest that prenatal exposure to BPA may affect child behavior, and differently among boys and girls.

Bisphenol A (BPA) is a weak estrogenic chemical used in the production of some plastics and resins (in food and drink containers), flame retardants, dental sealants, and in the recycling of thermal paper (Vandenberg et al. 2007). According to the National Health and Nutrition Examination Survey, the geometric mean urinary concentration of BPA in the general U.S. population sampled was 2.6 µg/L in 2003–2004; concentrations were significantly higher in women than in men, in children than in adults, and in individuals with low household income compared with individuals with higher income (Calafat et al. 2008).

BPA has been shown experimentally to interact with estrogen signaling pathways through binding to the estrogen receptors (Naciff et al. 2002; Vandenberg et al. 2009; Wetherill et al. 2007) and also to act as a thyroid hormone agonist (Zoeller et al. 2005). Prenatal exposure to BPA in mice led to alterations in brain structure and corresponding changes in behavior, with the latter being a notable end point for low dose exposure (reviewed by Chapin et al. 2008; Palanza et al. 2008). Alterations (elimination or reduction) of sexually dimorphic behaviors are a particularly sensitive end point for BPA exposure [reviewed by National Toxicology Program (2008)]. However, the experimental data are somewhat conflicting, with a number of animal studies reporting effects primarily in females and others mainly in males. Both sexes were not always tested, and different test systems and doses were used.

There are limited data on the neurodevelopmental effects of BPA exposure in humans. A recent epidemiological study found a positive association between maternal BPA urinary concentrations at gestational week 16 and externalizing behaviors, such as hyperactivity and aggression, at 2 years of age, with the association more pronounced in girls than in boys (Braun et al. 2009). Within the same cohort, at age 3 years, the mean maternal (16 and 26 weeks of gestation and birth) BPA urinary concentrations were associated with increased anxiety, depression, and hyperactivity among girls but not among boys (Braun et al. 2011b). In contrast, in a sample of 9-year-olds, their mothers’ third-trimester BPA urinary concentrations were not associated with symptoms of social impairment in both sexes combined, nor was there an interaction between prenatal BPA concentrations and child sex (Miodovnik et al. 2011).

In the present study we examined the association between prenatal adjusting for postnatal BPA exposure, estimated from BPA urinary concentrations, and behavior problems, which were measured by the Child Behavior Checklist (CBCL) (Achenbach and Rescorla 2001), in children 3–5 years of age. We hypothesized that prenatal exposure to BPA would be associated with symptoms of behavior problems after taking into consideration postnatal BPA exposure and covariates of interest. We postulated that there would be interactions between BPA exposure and sex of the child, as reported before (Braun et al. 2009, 2011b). Because the literature was not consistent on this point, we were agnostic as to the direction of effects within boys versus girls.

## Methods

*Sample selection.* Participants in this study were mothers and their children in the Columbia Center for Children’s Environmental Health (CCCEH) New York City (NYC) cohort. From 1998 through 2003, pregnant African-American and Dominican women who resided in Washington Heights, Harlem, or the South Bronx in New York City (USA) were recruited into a prospective cohort study (Perera et al. 2003). Inclusion was limited to women who were in the age range of 18–35 years, non-cigarette smokers, nonusers of other tobacco products or illicit drugs, free of diabetes, hypertension, or known HIV, and who initiated prenatal care by the 20th week of pregnancy. The Institutional Review Boards of the New York Presbyterian Medical Center and of the Centers for Disease Control and Prevention (CDC) approved the study; mothers provided informed consent for their children.

Three hundred sixty-one mother–child pairs had prenatal BPA measurements and maternal prenatal questionnaire data, and 337 children had CBCL data obtained at least once between the ages of 3 and 5 years (mean age, 3.6 years). The subset included in the present primary analysis was composed of 198 of these mother–child pairs who also had available data on all explanatory or potential confounding variables of interest as well as postnatal BPA measurements in the child at age 3 years.

*BPA measures.* Spot urine samples were collected from the mother during pregnancy (range, 24–40 weeks of gestation; mean, 34.0 weeks) and from the child between the ages of 3 and 4 years (range, 33–47 months; mean, 37.1 months). Previous research has indicated that the intraclass correlation coefficient for BPA urinary concentrations from multiple samples is low (Braun et al. 2011a) and within-day variance for spot collections is quite high (Ye et al. 2011). However, the ability of BPA concentrations from a single spot sample to predict exposure tertiles has been reported to be adequate (Mahalingaiah et al. 2008); and a separate study of minority children in NYC has shown that the BPA concentration in single samples is modestly predictive of the average 6-month concentration (Teitelbaum et al. 2008).

Immediately after collection, the urine samples were transported to the CCCEH laboratory, inventoried and frozen at –80°C before shipment to CDC for analysis. Total (free and conjugated) urinary concentrations of BPA were measured using online solid-phase extraction coupled with high-performance liquid chromatography–isotope dilution–tandem mass spectrometry with peak focusing (Ye et al. 2005). The limit of detection (LOD) was 0.4 µg/L; concentrations below the LOD were given a value of LOD/2 for the statistical analyses. Urinary dilution was calculated from specific gravity (SG) measurements obtained using a handheld refractometer (Urine-Specific-Gravity-Refractometer-PAL-10-S-P14643C0; TAGO USA, Inc., Bellevue, WA). All BPA concentrations were corrected for dilution by SG adjustment using a modification of the formula by Hauser et al. (2004). The formula *BPA_c_* = *BPA* × [(mean *SG* – 1)/(individual *SG* – 1)], where *BPA_c_* is the SG-corrected BPA concentration (micrograms per liter), *BPA* is the observed BPA concentration (micrograms per liter), and *SG* is the specific gravity of the urine sample (Hauser et al. 2004).

*Behavioral outcomes.* During a study visit when children were 3–5 years of age, research workers trained in neurodevelopmental testing oversaw the completion of the CBCL by the mothers, providing guidance as needed. The 99-item CBCL was completed in either English or Spanish depending on the mother’s language of choice. The CBCL consists of seven syndrome scales (Emotionally Reactive, Anxious/Depressed, Somatic Complaints, Withdrawn, Sleep Problems, Attention Problems, and Aggressive Behavior) and two composite scales, Internalizing Problems (sum of scores on Emotionally Reactive, Anxious/Depressed, Somatic Complaints, and Withdrawn) and Externalizing Problems (sum of scores on Attention Problems and Aggressive Behavior). The sum of the items of each CBCL syndrome scale provides a raw score denoting the extent of problems reported for the child on that scale, with higher scores corresponding to more problems. The CBCL is a well-validated measure of child behavior problems with good reliability and stability (Achenbach and Rescorla 2001).

*Statistical analysis.* For all analyses, both pre- and postnatal BPA concentrations (SG adjusted) were dichotomized at the upper quartile (high: upper quartile vs. low: other three quartiles). Because the original raw scores of each syndrome are count data that sum the scores on the specific items within each scale and the score distribution for each syndrome is right-skewed, we analyzed those scores using a Poisson regression. The two composite scales are normally distributed, so we analyzed them as continuous variables using linear regression. As an alternative we also converted the raw scores of the seven syndrome scales to *T*-scores to compare each child with a normative sample of children, and dichotomized the children in two outcome groups for each syndrome: borderline or clinical (*T*-score ≥ 65), or normal (*T*-score < 65) (Achenbach and Rescorla 2001). However, we did not analyze the dichotomous outcomes using logistic regression models because only a small percentage of children were classified as “borderline or clinical” according to this definition.

The analysis focused on BPA × sex interactions because previous studies have strongly suggested sex differences in BPA effects on neurodevelopment in both rats and humans (Braun et al. 2009; Poimenova et al. 2010). Therefore, we first examined the BPA × sex interaction on the seven CBCL syndrome scales and the two composite scales using Poisson and linear regression, respectively, with significant interactions defined based on interaction term *p*-values < 0.05. All parameter estimates and *p*-values were generated using SAS (version 9.1; SAS Institute Inc., Cary, NC). Although our analyses involved multiple comparisons, we did not perform the Bonferroni adjustment to reduce the possibility of making a type II error in this observational study (Rothman 1990).

Covariates were selected for the final model based on whether they were known or suspected risk factors according to the literature and our prior findings. These included sex of child (in models analyzing the entire set), gestational age of the child at birth (based on medical record data), mother’s intelligence score (measured with Test of Nonverbal Intelligence 3rd Edition, TONI-3) (Brown et al. 1990), mother’s completion of high school before the birth of the child (yes or no), mother’s demoralization status during pregnancy measured via the Psychiatric Epidemiology Research Instrument–Demoralization (PERI-D) scale (continuous scale of 0–4) (Dohrenwend et al. 1978), child’s age at CBCL assessment in months, the quality of the early home caretaking environment measured at 3 years of age via the HOME (Home Observation for Measurement of the Environment) Inventory (continuous measure) (Bradley 1994), prenatal exposure to environmental tobacco smoke (ETS) (any or none) in the home based on questionnaire, and postnatal BPA exposure, dichotomized at the upper quartile as high or low. In addition, prenatal phthalate exposure measured by urinary concentrations of phthalate metabolites during the third trimester of pregnancy (Whyatt et al. 2009) was correlated with prenatal BPA exposure (*r* = 0.153, *p* = 0.009). Therefore, we adjusted for SG-adjusted mono-*n*-butyl phthalate, which had the highest correlations of all phthalate metabolites with prenatal BPA concentrations and CBCL outcomes.

Finally, we evaluated prenatal polycyclic aromatic hydrocarbon (PAH) exposure, measured by personal monitoring during pregnancy (Perera et al. 2003), and postnatal exposure to ETS as potential confounders in a subset of children with these variables. Inclusion of these covariates did not materially influence the results, in that all significant associations present before adjustment remained and no additional significant associations emerged.

## Results

The sample included in this analysis consisted of 198 children who had complete data on CBCL and prenatal and postnatal BPA urinary concentrations and covariates; there were 87 boys and 111 girls. Table 1 compares characteristics of the study subset (*n* = 198) with those participants who had prenatal BPA measurements and maternal prenatal questionnaire data but who were not included because they were missing data on the CBCL or covariates (*n* = 159). Of the latter group, 24 children did not have a CBCL measurement completed within the age period of interest, 49 were missing data on covariates, and 86 were lacking postnatal BPA measurements. Demographic and exposure factors of the 198 children included in the analysis did not differ significantly from those of children who were not included because of missing data, except that the percentage of mothers reporting prenatal ETS exposure was higher in the group included in the analysis (34% vs. 23%). Furthermore, because most of the missing data were postnatal BPA measurements, we examined differences between the study sample (*n* = 198) and those excluded because of missing postnatal BPA only [*n* = 86; see Supplemental Material, Table S1 (http://dx.doi.org/10.1289/ehp.1104492)]. The two subsets were not significantly different in any of the variables considered except for gestational age (mean, 39.36 weeks for the subjects included, vs. 38.87 weeks for the subjects excluded, *p* = 0.004), nor do they differ by CBCL score (data not shown).

**Table 1:** Comparison of the characteristics of children included and excluded from BPA–CBCL analyses.

Variable	Subjects in the analysis (n = 198)	Subjects not included^a^ (n = 159)	n	p-Value
Prenatal BPA urinary concentration (μg/L)		1.96 (3.12)b		2.09 (5.20)b		159		0.089
3-year BPA urinary concentration (μg/L)		3.94 (10.93)b		4.29 (8.22)b		54		0.894
Prenatal mono-n-butyl phthalate concentration (μg/L)		63.42 ± 97.34		64.92 ± 77.56		146		0.874
Age at assessment (months)		38.27 ± 5.24		37.98 ± 6.84		142		0.673
Percent with prenatal ETS exposure		33.84		22.64		159		0.020
Percent female		56.06		49.69		159		0.230
Percent ≥ high school education		58.59		63.52		159		0.342
Percent African American		36.87		32.70		159		0.412
Gestational age at birth (weeks)		39.36 ± 1.25		39.19 ± 1.30		159		0.210
Maternal TONI score		19.83 ± 8.94		19.88 ± 7.99		131		0.958
HOME inventory		39.69 ± 6.34		39.10 ± 6.18		134		0.401
Maternal demoralization score		1.09 ± 0.63		1.12 ± 0.64		157		0.693
Values are mean ± SD or percent. aA total of 361 children had prenatal BPA measurements and maternal prenatal questionnaire data. Some subjects were not included in the analysis due to missing CBCL data (n = 24), measurement of postnatal BPA (n = 86), or missing information on at least one other covariate (n = 49). bThe means for prenatal and 3-year BPA are geometric means.								

The distribution of CBCL scores for children included in the analysis is shown in Table 2. The observed raw scores ranged from 0 to 42. The boys and girls did not differ significantly in mean score or percentage of children in the borderline or clinical range. The distribution of CBCL scores in our study is similar to that for other low-income populations (Gross et al. 2006).

**Table 2:** Distribution of CBCL raw score outcomes in the cohort of 198 children.

Boys (n = 87)					Girls (n = 111)				
CBCL	Score range	Mean of scores	n (%) in borderline or clinical rangea	Score range	Mean of scores	n (%) in borderline or clinical range
Syndrome scores												
Emotionally Reactive		0–11		2.08		9 (10.34)		0–9		2.08		8 (7.21)
Anxious/Depressed		0–13		3.23		10 (11.49)		0–12		3.36		11 (9.91)
Somatic Complaints		0–12		2.36		13 (14.94)		0–9		2.29		18 (16.22)
Withdrawn		0–8		1.93		11 (12.64)		0–13		2.02		16 (14.41)
Sleep Problems		0–11		2.68		3 (3.45)		0–11		3.1		7 (6.31)
Attention Problems		0–8		3.03		10 (11.49)		0–7		2.72		7 (6.31)
Aggressive Behavior		0–35		10.36		5 (5.75)		0–31		10.27		12 (10.81)
Composite scores												
Internalizing Problems		0–38		9.6		11 (12.64)		0–28		9.75		10 (9.01)
Externalizing Problems		0–42		13.39		6 (6.90)		0–37		12.99		11 (9.91)
aWe converted the raw scores of the seven syndrome scales to T-scores to compare each child to a normative sample of children, and dichotomized the children in two outcome groups for each syndrome: borderline or clinical (T-score ≥ 65), or normal (T-score < 65). The Internalizing and Externalizing scales are not truncated because very few children have extremely low scores owing to the large number of problem items on these composite scales (Achenbach and Rescorla 2001).												

Table 3 shows the distribution of BPA concentrations by percentiles in urine samples collected from mothers during pregnancy and from their children at 3 years of age. BPA was detectable in the urine of > 90% of both mothers and children. The ranges of total SG-adjusted BPA urinary concentrations in maternal and child urine samples were 0.24–38.53 µg/L and 0.42–73.50 µg/L, respectively, indicating a wide variation in exposure. Geometric means for maternal and child urinary concentrations of BPA were 1.96 and 3.94 µg/L, respectively. There were no significant differences between boys and girls in median and mean prenatal or postnatal BPA concentrations, whether SG-adjusted or not.

**Table 3:** Descriptive statistics of prenatal and postnatal BPA concentrationsa among 87 boys and 111 girls included in the analyses.

Percentile							Geometric mean
Time of BPA collection	Minimum	5th	25th	50th	75th	95th		Maximum	Mean
Prenatal BPA										
No specific gravity adjustment (μg/L)										
All children	< LOD	0.40	1.10	1.80	3.10	8.50	30.00	2.68		1.80
Girls	< LOD	< LOD	1.00	1.90	3.20	9.20	30.00	3.01		1.84
Boys	< LOD	0.40	1.20	1.75	3.00	5.80	8.50	2.24		1.75
Specific gravity adjusted (μg/L)										
All children	0.24	0.66	1.23	1.96	3.04	7.16	38.53	2.76		1.96
Girls	0.24	0.24	1.17	2.02	3.20	7.65	31.87	2.87		2.03
Boys	0.28	0.72	1.23	1.82	2.73	5.67	38.53	2.61		1.88
3-year BPA										
No specific gravity adjustment (μg/L)										
All children	< LOD	0.60	1.70	3.55	6.40	27.60	90.80	6.86		3.56
Girls	< LOD	< LOD	1.40	3.10	8.00	31.20	62.80	6.97		3.45
Boys	< LOD	0.70	2.35	3.95	6.15	19.80	90.80	6.80		3.71
Specific gravity adjusted (μg/L)										
All children	0.42	1.11	2.24	3.19	7.08	20.10	73.50	6.17		3.94
Girls	0.42	0.42	2.21	3.22	7.77	20.74	42.65	6.31		4.01
Boys	0.68	1.08	2.30	3.19	6.37	14.96	73.50	6.05		3.86
aPrenatal BPA concentrations were analyzed in maternal urine samples taken during the third trimester. Postnatal BPA concentrations were analyzed in child urine samples taken when the child was 3 years of age.										

Significant interactions (*p* < 0.05) were observed between prenatal BPA urinary concentrations (adjusting for postnatal BPA exposure and other covariates) and sex on Emotionally Reactive, Aggressive Behavior, and Internalizing Problems (Table 4). After stratifying on sex, the BPA effects were positive and significant among boys on Emotionally Reactive and Aggressive Behavior and positive and borderline significant (*p* < 0.1) on Sleep Problems, Withdrawn, Internalizing Problems, and Externalizing Problems, indicating that boys with prenatal BPA exposure in the highest concentration quartile had, on average, more reported symptoms of problems in these areas. For the syndrome of Emotionally Reactive for example, symptoms scores were 1.62 times higher [95% confidence interval (CI): 1.13,2.32] among boys with high prenatal BPA concentrations compared with those with low prenatal BPA concentrations. In contrast, among girls, high BPA exposure was associated with lower scores for Anxious/Depressed and Aggressive Behavior (*p* < 0.05), and Internalizing Problems (*p* < 0.1) indicating that girls in the high prenatal BPA exposure group had, on average, fewer reported problems in these areas than girls in the low exposure group. Postnatal BPA urinary concentration alone had a significant negative effect only on Emotionally Reactive within the entire sample [see Supplemental Material, Table S2 (http://dx.doi.org/10.1289/ehp.1104492)]. Comparison of results before and after adjusting for postnatal BPA exposure found the effect estimates to be similar.

**Table 4:** Association between prenatal BPA (high/low) and CBCL scores, adjusting for postnatal BPA and covariates.

All children (n = 198)			Boys (n = 87)			Girls (n = 111)			Interaction (n = 198) p-Value
Estimate (95% CI)	p-Value	Estimate (95% CI)	p-Value	Estimate (95% CI)	p-Value				
Syndrome scores														
Emotionally Reactive		1.03 (0.8, 1.32)		0.825		1.62 (1.13, 2.32)		0.008		0.74 (0.51, 1.07)		0.112		0.002
Anxious/Depressed		0.95 (0.78, 1.16)		0.627		1.23 (0.91, 1.67)		0.175		0.75 (0.57, 0.99)		0.040		0.083
Somatic Complaints		0.93 (0.74, 1.18)		0.574		1.16 (0.82, 1.64)		0.412		0.77 (0.55, 1.09)		0.139		0.123
Withdrawn		1.02 (0.8, 1.31)		0.860		1.42 (0.97, 2.07)		0.072		0.79 (0.56, 1.11)		0.177		0.068
Sleep Problems		1.08 (0.88, 1.33)		0.437		1.38 (1.00, 1.90)		0.051		0.92 (0.7, 1.21)		0.562		0.077
Attention Problems		0.98 (0.79, 1.21)		0.826		1.24 (0.90, 1.70)		0.192		0.80 (0.59, 1.08)		0.149		0.137
Aggressive Behavior		0.99 (0.88, 1.11)		0.827		1.29 (1.09, 1.53)		0.003		0.82 (0.70, 0.97)		0.017		0.001
Composite scores														
Internalizing Problems		–0.06 (–2.29, 2.18)		0.961		3.28 (–0.42, 6.97)		0.083		–2.35 (–5.02, 0.31)		0.084		0.037
Externalizing Problems		–0.18 (–2.97, 2.6)		0.898		3.53 (–0.45, 7.5)		0.082		–2.51 (–6.31, 1.29)		0.195		0.076
Pre- and postnatal BPA concentrations were dichotomized as upper quartile based on logarithm-transformed SG-adjusted values. Four types of models were fitted separately to assess the main effects of prenatal BPA on both boys and girls, boys only, and girls only and the interaction effects of prenatal BPA on both boys and girls. The covariates in each model are the same: postnatal BPA measurements, prenatal mono-n-butyl phthalate concentration, age at assessment (in months), smoking at home (yes or no), child sex, maternal education (≥ high school or not), ethnicity (African American or not), gestational age (in weeks), HOME inventory score, TONI score, and PERI-D score. The seven syndromes scales were fitted using Poisson log-linear models. For interpretability we report the exponentiated beta in the estimate column. The two composite scales were fitted in linear models. Therefore, in that estimate column, the values are the original betas. In addition, the estimates for the syndrome scores indicate multiplicative difference between high and low exposure, whereas the estimate for composite scores indicates the average difference in scores for high versus low exposure on an absolute scale.														

In sensitivity analyses, we noticed that one male child had among the highest prenatal BPA concentrations as well as several CBCL scores and could be an influential variable. As shown in Supplemental Table S4 (http://dx.doi.org/10.1289/ehp.1104492), removal of this subject from the analysis reduced the beta and *p*-values in general but did not alter the direction of the associations. The BPA × sex interactions remained significant for Emotionally Reactive and Aggressive Behavior but not for Internalizing Problems.

## Discussion

In this longitudinal cohort study we examined the association between prenatal BPA exposure and child behavior in preschool-age children, accounting for postnatal BPA and other potential confounders. Among boys, prenatal BPA exposure was positively associated with higher scores on all syndromes and significantly associated with Emotionally Reactive and Aggressive Behavior. Inverse associations were seen in girls for all syndromes and these associations were significant for Anxious/Depressed and Aggressive Behavior.

The present finding that prenatal exposure to BPA is associated with more symptoms of certain behavioral problems in 3- to 5-year-old boys is consistent with some, but not all, prior studies in laboratory animals. Some animal studies have shown that in male mice, prenatal BPA exposure is associated with increased aggression (Kawai et al. 2003) and with memory impairment and hyperactivity (no female offspring were tested) (Miyagawa et al. 2007; Mizuo et al. 2004). Others have reported changes in behavior or related pathways have included greater anxiety-like behavior in female mice (male mice were not tested) (Ryan and Vandenbergh 2006), changes in the dopaminergic and *N*-methyl-d-aspartate-ergic (NMDA) systems (associated with memory and behavior) (Tian et al. 2010), and reduction of activity and desire to explore in both sexes (Farabollini et al. 1999). In addition, mice of both sexes exposed to low doses of BPA prenatally (20 µg/kg of BPA daily from embryonic day until postnatal day 21) and throughout lactation had an increase in serotonin and dopamine (involved in mood, memory, and learning) levels in certain brain regions compared with a control group not exposed to BPA (Nakamura et al. 2010). In one study of rats, maternal BPA exposure inhibited the protein expression of NMDA receptor subunits and estrogen receptor beta of the hippocampus of male offspring during postnatal development, suggesting that BPA may affect the male developing brain; data on females were not reported (Xu et al. 2010). Rats prenatally exposed to BPA have an increase in anxiety-like behavior and a decrease in desire to explore, with the effects more pronounced in females than in males (Poimenova et al. 2010). BPA has also been found to abrogate sexual dimorphism in brain structure and behavior in rats (determined by estrogen signaling) (McCarthy 2008) and to disrupt cognition, social behaviors, and other aspects of brain function (Richter et al. 2007).

Male cynomolgus monkeys who were prenatally exposed to BPA behaved like non-BPA exposed female infants rather than non-BPA exposed males, suggesting that BPA affects behavior and sexual differentiation in male monkeys (Nakagami et al. 2009). Although interspecies variation in internal dose and response to BPA is a concern (Doerge et al. 2010), BPA pharmacokinetics appear to be similar in women, female monkeys, and mice (Taylor et al. 2011).

Our findings are inconsistent with reports by Braun et al. (2009, 2011b). Although these investigators also examined sex-specific effects of prenatal BPA exposure on some dimensions of child behavior, they reported evidence of adverse effects predominantly in girls. Specifically, Braun et al. found a relationship between prenatal BPA exposure and externalizing behavior in 2-year-old girls but not boys using the Behavioral Assessment System for Children (BASC) (Braun et al. 2009). At the age 3 years follow-up, they also found that gestational BPA concentrations were associated with anxiety, depression, and hyperactivity among girls but not boys in their population. Including childhood BPA concentrations in the models did not significantly change the results (Braun et al. 2011b). They collected maternal spot urine samples twice during pregnancy and at delivery, with urinary concentrations of BPA being similar to those in the present study (median concentrations of 1.8, 1.7, and 1.3 µg/L at 16 and 26 weeks of pregnancy and at birth, respectively). Child spot urine samples were collected at 1, 2, and 3 years of age. The 16-week BPA (early second trimester) prenatal urinary concentrations were more highly correlated with behavioral outcomes than were the prenatal 26-week (early third trimester) or birth BPA measurements. We note that when we compare the results of Braun et al. for urinary BPA measures at 26 weeks and at birth [i.e., the measurement times closest to our prenatal measurement (34.0 weeks on average)], both boys and girls had lower (i.e., better) scores on Internalizing and Externalizing scales, though the results were not statistically significant.

Although these would not fully explain the discrepant findings, there were a number of differences between the study designs, especially the differences in the timing of sample collection. We measured BPA at a single time point in the third trimester of pregnancy, whereas Braun et al. (2011b) took three serial measurements. In their analysis the prenatal exposure with the greatest associations with child behavior at age 2 and 3 years was measured in samples taken at 16 weeks, with BPA measurements taken later in pregnancy having weaker associations. In addition, the children in the CCCEH cohort at assessment were older (3–5 years vs. 2 and 3 years) than in those studied by Braun et al. (2011b). Also, different instruments were used to assess child behavior, and different statistical models were used to analyze the data. The children in the two study populations also differed by both ethnicity and socioeconomic status. Specifically, our population is a low-income minority population, whereas most of the participants included in the Braun study were white with a household income of > $40,000. Finally, prior research has shown that the two instruments (CBCL and BASC-2) give similar, but not identical, results (Myers et al. 2010). Because the mechanisms of the association between early BPA exposure and child behavior are currently not understood, further research is needed to identify the factors that influence the expression of BPA behavioral toxicity in children.

A strength of our study is the ability to control for a number of variables based on medical record, questionnaire, biomarker, and air monitoring data that may affect neurobehavioral development. Because we measured BPA concentrations in child urine, we were able to compare results before and after adjusting for postnatal BPA exposure. The finding that the results were similar before and after adjusting for postnatal exposure suggests that the prenatal period may be a more sensitive window for BPA exposure [see Supplemental Material, Tables S2, S3 (http://dx.doi.org/10.1289/ehp.1104492)]. In addition, we were able to adjust for prenatal phthalate exposure as a potential confounder. A limitation of our study is the use of a single measurement of BPA in urine to categorize exposure. Data are lacking on the time points during fetal development that are most susceptible to BPA. It would have been preferable to have had multiple BPA measures in repeated urine samples over the pregnancy and during childhood. Although we recognize the limitation of using a single spot urine sample as a measurement of chronic BPA exposure (Braun et al. 2011a), we would expect the noise in measurements to bias associations toward the null. Another limitation of this study is the relatively small sample size (*n* = 198), particularly for assessing interactions by sex. In addition, because our research focused on minority women and children living in an urban setting, the effects may not be generalizable to other racial and ethnic groups, nor to other exposed populations. However, the distribution of CBCL scores in our study is similar to that for other low-income populations (Gross et al. 2006).

Additionally, because the CBCL is a parent account of child behavior, it is subject to limitations inherent in such instruments including reporting bias or a difficulty inferring a child’s internal state. Finally, although we adjusted for other exposures and potential confounders, there is the possibility of residual confounding by unmeasured exposures or other stressors.

In conclusion, we report an association between prenatal BPA exposure and child behavior at age 3–5 years, with sex-specific associations. Among boys we found significant positive associations between prenatal BPA and CBCL scores of Emotionally Reactive and Aggressive Behavior. In contrast, among girls prenatal BPA was associated with significantly lower scores for the Aggressive Behavior and Anxious/Depressed syndromes.

Follow-up of the cohort is ongoing to further evaluate these sex-specific associations at older ages. Behavioral problems such as those observed here may be of concern for future academic performance and social functioning (Hinshaw 1992; Wood 2006).

## Supplemental Material

(106 KB) PDF
