# Supplementary material for: Prenatal Bisphenol A Exposure and Child Behavior in an Inner-City Cohort
Source: Environ Health Perspect. 2012 Apr 27;120(8):1190–4. doi: 10.1289/ehp.1104492 (PMC3440080; doi:10.1289/ehp.1104492)
Supplement: (106 KB) PDF [file ehp.1104492.s001.pdf]

Supplemental Material

Prenatal Bisphenol A Exposure and Child Behavior in an Inner City Cohort

Frederica Perera, Julia Vishnevetsky, Julie B. Herbstman, Antonia M. Calafat, Wei Xiong, Virginia Rauh, Shuang Wang

Table of Contents

Supplemental Material, Table S1. Comparison between the study sample included and sample missing postnatal BPA concentrations. 3

Supplemental Material Table S2. Quartile postnatal BPA on CBCL scores adjusting for covariates and quartile prenatal BPA[a,b, c] 4

Supplemental Material, Table S3. Association between quartile prenatal BPA concentrations and CBCL scores, adjusting for covariates and quartile prenatal BPA [a, b, c] 5

Supplemental Material Table S4. Association between prenatal BPA (high/low) and CBCL scores, adjusting for postnatal BPA and covariates, excluding one male subject [a, b, c , d] 6

Supplemental Material, Table S1. Comparison between the study sample included and sample missing postnatal BPA concentrations.

	Subjects in the analysis (N=198)		Subjects not included[a] (N=86)		P-value for testing difference
Variable	Mean	Std Dev	Mean	Std Dev	
Prenatal BPA urinary concentration (μg/L)	1.96 [b]	3.12	2.14 [b]	6.40	0.163
Prenatal mono-n-butyl phthalate urinary concentration (μg/L)	63.42	97.34	60.23	76.59	0.767
Age at assessment (month)	38.27	5.24	38.62	6.02	0.623
% smoking at home	33.84%		24.42%		0.115
% female	56.06%		48.84%		0.262
%≥ high school education	58.59%		62.79%		0.507
% African American	36.87%		32.56%		0.486
Gestational age (weeks)				1.36	0.004
Maternal TONI score	19.83	8.94	19.17	8.22	0.562
Home environment	39.69	6.34	39.78	5.72	0.913
Maternal demoralization score	1.09	0.63	1.12	0.62	0.667

[a]Subjects with CBCL scores, prenatal BPA measurements and all other covariates except postnatal BPA

[b] The means of prenatal BPA are geometric means.

Supplemental Material Table S2. Quartile postnatal BPA on CBCL scores adjusting for covariates and quartile prenatal BPA[a,b, c]

	All children (N = 198)		Boys (N = 87)		Girls (N = 111)		Interaction (N=198)
CBCL outcome	Estimate (95% CI)	p-value	Estimate (95% CI)	p-value	Estimate (95% CI)	p-value	p-value
Emotionally Reactive	0.76 (0.59, 0.97)	0.029	0.94 (0.63, 1.4)	0.764	0.74 (0.53, 1.03)	0.078	0.474
Anxious/ Depressed	1.08 (0.9, 1.3)	0.403	1.23 (0.92, 1.67)	0.162	1.14 (0.9, 1.46)	0.274	0.715
Somatic Complaints	1.01 (0.81, 1.26)	0.913	0.92 (0.64, 1.34)	0.679	1.12 (0.84, 1.49)	0.445	0.379
Withdrawn	0.99 (0.79, 1.26)	0.956	1.01 (0.68, 1.49)	0.958	1.08 (0.79, 1.48)	0.615	0.651
Sleep problems	1.08 (0.9, 1.31)	0.428	0.93 (0.66, 1.3)	0.661	1.22 (0.95, 1.55)	0.113	0.200
Attention Problems	0.88 (0.72, 1.08)	0.216	0.98 (0.71, 1.34)	0.891	0.85 (0.64, 1.13)	0.271	0.766
Aggressive Behavior	0.93 (0.84, 1.04)	0.201	1.03 (0.87, 1.22)	0.748	0.91 (0.79, 1.05)	0.207	0.803
	All children (N = 198)		Boys (N = 87)		Girls (N = 111)		Interaction (N=198)
CBCL outcome	Estimate (95% CI)	p-value	Estimate (95% CI)	p-value	Estimate (95% CI)	p-value	p-value
Internalizing Problems	-0.20 (-2.25, 1.86)	0.851	0.37 (-2.90, 3.64)	0.823	0.27 (-2.22, 2.76)	0.830	0.821
Externalizing Problems	-1.02 (-3.57, 1.54)	0.435	0.16 (-3.34, 3.67)	0.928	-1.39 (-4.92, 2.14)	0.439	0.889

[a] Pre- and postnatal BPA urinary concentrations were SG-adjusted and dichotomized as Q4 vs. Q1-Q3 based on logarithm transformed SG adjusted values

[b] Adjusted for postnatal BPA measurements, prenatal mono-n-butyl phthalate concentration, age at assessment, smoking at home, child sex, maternal education (≥high school or not), ethnicity (African American or not), gestational age, HOME inventory, TONI score, and maternal demoralization during pregnancy.

[c] The seven Syndromes scales were fitted using Poisson log-linear models. For interpretability we report the exponentiated beta in the Estimate column. The two Composite scales were fitted in linear models. Therefore, in that Estimate column, the values are the original betas. In addition, the estimates for the Syndrome scores indicate multiplicative difference between high and low exposure, whereas the estimate for composite scores indicates the average difference in scores for high versus low exposure on an absolute scale

Supplemental Material, Table S3. Association between quartile prenatal BPA concentrations and CBCL scores, adjusting for covariates and quartile prenatal BPA [a, b, c]

	All children (N = 198)		Boys (N = 87)		Girls (N = 111)		Interaction (N=198)
CBCL outcome	Estimate (95% CI)	p-value	Estimate (95% CI)	p-value	Estimate (95% CI)	p-value	p-value
Emotionally Reactive	1.01 (0.78, 1.29)	0.959	1.62 (1.14, 2.32)	0.008	0.71 (0.50, 1.02)	0.064	0.001
Anxious/ Depressed	0.96 (0.79, 1.17)	0.691	1.22 (0.90, 1.66)	0.195	0.77 (0.58, 1.01)	0.060	0.102
Somatic Complaints	0.94 (0.74, 1.19)	0.579	1.16 (0.82, 1.64)	0.401	0.79 (0.57, 1.11)	0.171	0.128
Withdrawn	1.02 (0.80, 1.31)	0.864	1.42 (0.97, 2.07)	0.072	0.80 (0.57, 1.13)	0.199	0.069
Sleep problems	1.09 (0.89, 1.34)	0.395	1.38 (1.00, 1.90)	0.051	0.95 (0.73, 1.25)	0.719	0.089
Attention Problems	0.97 (0.78, 1.20)	0.744	1.24 (0.90, 1.70)	0.191	0.78 (0.58, 1.06)	0.110	0.115
Aggressive Behavior	0.98 (0.88, 1.10)	0.732	1.29 (1.09, 1.53)	0.003	0.81 (0.69, 0.95)	0.008	0.001
	All children (N = 198)		Boys (N = 87)		Girls (N = 111)		Interaction (N=198)
CBCL outcome	Estimate (95% CI)	p-value	Estimate (95% CI)	p-value	Estimate (95% CI)	p-value	p-value
Internalizing Problems	-0.09 (-2.32, 2.14)	0.937	3.28 (-0.42, 6.98)	0.082	-2.33 (-4.97, 0.31)	0.084	0.035
Externalizing Problems	-0.29 (-3.07, 2.49)	0.837	3.52 (-0.45, 7.49)	0.082	-2.73 (-6.50, 1.05)	0.157	0.069

[a] Pre- and postnatal BPA urinary concentrations were SG-adjusted and dichotomized as Q4 vs. Q1-Q3 based on logarithm transformed SG adjusted values

[b] Adjusted for postnatal BPA measurements, prenatal mono-n-butyl phthalate concentration, age at assessment, smoking at home, child sex, maternal education (≥high school or not), ethnicity (African American or not), gestational age, HOME inventory, TONI score, and maternal demoralization during pregnancy.

[c] The seven Syndromes scales were fitted using Poisson log-linear models. For interpretability we report the exponentiated beta in the Estimate column. The two Composite scales were fitted in linear models. Therefore, in that Estimate column, the values are the original betas. In addition, the estimates for the Syndrome scores indicate multiplicative difference between high and low exposure, whereas the estimate for composite scores indicates the average difference in scores for high versus low exposure on an absolute scale

Supplemental Material Table S4. Association between prenatal BPA (high/low) and CBCL scores, adjusting for postnatal BPA and covariates, excluding one male subject [a, b, c , d]

	All children (N = 197)		Boys (N = 86)		Girls (N = 111)		Interaction (N = 197)
Syndrome Scores	Estimate (95% CI)	p-value	Estimate (95% CI)	p-value	Estimate (95% CI)	p-value	p-value
Emotionally Reactive	1.27 (0.87, 1.87)	0.220	1.37 (0.92, 2.03)	0.118	0.74 (0.51, 1.07)	0.112	0.037
Anxious/ Depressed	1.00 (0.73, 1.39)	0.977	1.07 (0.77, 1.50)	0.684	0.75 (0.57, 0.99)	0.040	0.371
Somatic Complaints	1.13 (0.79, 1.62)	0.493	1.14 (0.79, 1.64)	0.494	0.77 (0.55, 1.09)	0.139	0.145
Withdrawn	1.17 (0.79, 1.75)	0.437	1.24 (0.83, 1.87)	0.295	0.79 (0.56, 1.11)	0.177	0.231
Sleep problems	1.35 (0.97, 1.87)	0.071	1.36 (0.98, 1.90)	0.068	0.92 (0.7, 1.21)	0.562	0.090
Attention Problems	1.10 (0.80, 1.53)	0.549	1.18 (0.84, 1.64)	0.337	0.80 (0.59, 1.08)	0.149	0.236
Aggressive Behavior	1.14 (0.96, 1.36)	0.145	1.19 (1.00, 1.43)	0.052	0.82 (0.70, 0.97)	0.017	0.010
	All children (N = 197)		Boys (N = 86)		Girls (N = 111)		Interaction (N = 197)
Composite Scores	Estimate (95% CI)	p-value	Estimate (95% CI)	p-value	Estimate (95% CI)	p-value	p-value
Internalizing Problems	1.32 (-2.16, 4.80)	0.457	1.88 (-1.65, 5.41)	0.296	-2.35 (-5.02, 0.31)	0.084	0.160
Externalizing Problems	1.81 (-2.65, 6.26)	0.427	2.42 (-1.50, 6.35)	0.226	-2.51 (-6.31, 1.29)	0.195	0.174

[a] Pre- and postnatal BPA concentrations were dichotomized as upper quartile based on logarithm transformed SG-adjusted values

[b] Four types of models were fitted separately to assess the main effects of prenatal BPA on both boys and girls (column 1), boys only (column 2) and girls only (column 3) and the interaction effects of prenatal BPA on both boys and girls (column 4).

[c] The covariates in each model are the same: postnatal BPA measurements, prenatal mono-n-butyl phthalate concentration, age at assessment (in months), smoking at home (yes or no), child sex, maternal education (≥high school or not), ethnicity (African American or not), gestational age (in weeks), HOME inventory score, TONI score, and PERI-D score.

[d] The seven Syndromes scales were fitted using Poisson log-linear models. For interpretability we report the exponentiated beta in the Estimate column. The two Composite scales were fitted in linear models. Therefore, in that Estimate column, the values are the original betas. In addition, the estimates for the Syndrome scores indicate multiplicative difference between high

and low exposure, whereas the estimate for composite scores indicates the average difference in scores for high versus low exposure on an absolute scale
